# Reply: Implications of presymptomatic change in thalamus and caudate in Alzheimer’s disease

**DOI:** 10.1093/brain/awt168

**Published:** 2013-07-03

**Authors:** Natalie S. Ryan, Nick C. Fox

**Affiliations:** Dementia Research Centre, Department of Neurodegenerative Disease, University College London (UCL) Institute of Neurology, Queen Square, London, WC1N 3BG, UK



Sir, We thank Dr Vishnu for his interest in our paper. We reported atrophy of the caudate and thalamus in presymptomatic familial Alzheimer’s disease mutation carriers at a stage when hippocampal atrophy was not yet evident (Ryan *et al.*, 2013). As Dr Vishnu (2013) describes, brain atrophy on MRI is thought to be a biomarker of neuronal loss, which is considered to be a downstream element in the amyloid cascade hypothesis of Alzheimer’s disease pathogenesis. Dr Vishnu raises the question of whether the caudate and thalamic atrophy we detected represents neuronal injury induced by amyloid or by amyloid-independent mechanisms. We agree that this is an important point and feel that it in fact highlights an even broader issue; that it is not yet clear what pathological processes do account for the volume or other MRI-based changes that may be witnessed in the presymptomatic phase of Alzheimer’s disease. Although there are numerous clinicopathological studies correlating atrophy on MRI with neuronal loss, Braak neurofibrillary tangle stage and tau burden in patients with established symptomatic Alzheimer’s disease (Zarow *et al.*, 2005; Whitwell *et al.*, 2008), this information is lacking for the presymptomatic stage. Various different processes may give rise to changes in the volume of brain structures, some of which may be dynamic, and this uncertainty should be taken into account in hypothetical biomarker models of presymptomatic Alzheimer’s disease.

We proposed in our article (Ryan *et al.*, 2013) that axonal injury and subsequent degeneration may account for the thalamic and caudate atrophy that we observed in the presymptomatic mutation carriers. Support for this hypothesis came from the associated changes in diffusivity indices that we found in both of these subcortical grey matter structures and also in the cingulum. Interestingly, the same pattern of diffusivity changes that we detected in our presymptomatic mutation carriers has also been observed in a diffusion tensor imaging study of the APPsw transgenic mouse at the time of amyloid plaque accumulation (Sun *et al.*, 2005). The fact that this mouse model of Alzheimer’s disease does not develop tau pathology perhaps indirectly supports the idea that neuronal injury in presymptomatic familial Alzheimer’s disease may be induced by amyloid pathology alone.

Some support for the idea that processes other than overt neuronal loss may account for the thalamic atrophy evident on MRI comes from one of the few autopsy studies to have specifically examined the thalamus in patients with Alzheimer’s disease. Xuereb *et al.* (1991) noted that, although there was significant loss of thalamic volume in cases with Alzheimer’s disease, the amount of neuronal loss was insufficient to account for degree of atrophy. They hypothesized that the atrophy must instead be due to loss of axons, dendrites and synaptic structures or to glial cell changes. In our paper (Ryan *et al.*, 2013), we focused on the potential role that axonal degeneration may play in the development of subcortical atrophy. However, it is also important to consider the possibility that glial cell changes may contribute to the volumetric MRI changes evident in presymptomatic familial Alzheimer’s disease. Studies in a triple transgenic mouse model of Alzheimer’s disease have revealed complex changes in astroglial morphology during the early stages of the disease (Olabarria *et al.*, 2010). Before the appearance of neuritic amyloid plaques, hippocampal astrocytes have been observed to undergo atrophy, but once the plaques arise, those in close vicinity become gliotic whilst those further away remain atrophied. One can envisage that altering glial numbers or morphology might be reflected in dynamic changes in the volume and diffusion characteristics of the affected brain structures when studied with MRI at different time-points in the presymptomatic stage.

If immune-mediated mechanisms do play a role that is reflected in dynamic regional brain volume changes this could, as Dr Vishnu notes, explain Fortea *et al.*’s (2010) contrasting findings of caudate enlargement in presymptomatic familial Alzheimer’s disease mutation carriers. However, drawing comparisons between different studies of presymptomatic familial Alzheimer’s disease is difficult for a number of reasons. Not only do they vary in how far from expected age at symptom onset the subjects in the study are, they often use different measures to define estimated age at onset. These include parental age at onset or the mean or median age at onset for the family. It is not yet clear which of these predictors is most accurate, nor how much natural variability in age at onset may be expected within a family. The subjective nature of deciding what constitutes the onset of ‘symptoms’ and variability in how this is defined for the purpose of a study further complicates matters (Ryan and Rossor, 2011). Finally, different techniques for segmenting a structure of interest like the caudate on MRI may be employed by different studies and consistency between methods has not been systematically evaluated.

We agree with Dr Vishnu (2013) that studying mutation carriers with a variety of biomarkers including amyloid imaging and CSF measures of amyloid and tau, in addition to MRI, will be crucial to understanding the mechanisms operating in presymptomatic Alzheimer’s disease. Fortunately, initiatives like the Dominantly Inherited Alzheimer Network and the Colombian Alzheimer’s Prevention Initiative Registry are currently collecting such data in large cohorts of presymptomatic individuals and cross-sectional analyses have already provided insights into the probable sequence of biomarker changes (Bateman *et al.*, 2012; Reiman *et al.*, 2012). Ultimately however, it is likely to be the longitudinal analysis of multiple time-point multimodal data and ascertainment of rates of change that will reveal the most information about the underlying pathological mechanisms. Importantly, trials of disease-modifying therapies for presymptomatic familial Alzheimer’s disease will soon be launched and will include at least some assessment of biomarker changes (Bateman *et al.*, 2011). Biomarkers have the potential to behave in unexpected ways following treatment, as illustrated by the AN1792 amyloid-β active immunization trial, in which it was the antibody-responders who showed the greatest rates of atrophy (Fox *et al.*, 2005). Given that immune mechanisms may play a role in both the early disease process, and in the strategies used to combat it with amyloid-immunomodulatory agents, efforts should be made to better understand how such processes affect imaging biomarkers. A variety of imaging techniques may play a role here including microglial activation studies, as may the insights gained from imaging of animal models. Much valuable work has been done to formulate hypothetical models of Alzheimer’s disease biomarkers using evidence gathered from studies of sporadic Alzheimer’s disease, mild cognitive impairment and normal ageing (Jack *et al.*, 2013). The challenge now is to use longitudinal studies of presymptomatic mutation carriers to better understand the temporal evolution of biomarker changes during the natural history of familial Alzheimer’s disease, so that these models may be further refined and the optimal time for therapeutic intervention may be guided.

## Funding

This work was supported by the Medical Research Council (Clinical Research Training Fellowship G0900421 to NSR, Senior Clinical Fellowship G116/143 to NCF). It was undertaken at UCLH/UCL, who received a proportion of funding from the Department of Health's National Institute for Health Research (NIHR) Biomedical Research Centres funding scheme, and was supported by the NIHR Queen Square Dementia Biomedical Research Unit. NCF is a NIHR senior investigator. The Dementia Research Centre is an Alzheimer's Research UK Co-ordinating Centre and has received equipment funded by Alzheimer's Research UK and Brain Research Trust.
